# Ontological model of multi-agent Smart-system for predicting drug properties based on modified algorithms of artificial immune systems

**DOI:** 10.1186/s12976-020-00130-x

**Published:** 2020-07-20

**Authors:** Galina Samigulina, Zarina Samigulina

**Affiliations:** 1 Institute of Information and Computing Technologies, Lab.«Intellectual Control Systems and Forecasting», Almaty, Kazakhstan; 2 Faculty of Information Technologies, Kazakh-British Technical University, Almaty, Kazakhstan

**Keywords:** Smart-system, Drug design, Artificial immune system, Ontological model, Modified algorithms, Multi-agent system

## Abstract

**Background:**

Currently, due to the huge progress in the field of information technologies and computer equipment, it is important to use modern approaches of artificial intelligence in order to process extensive chemical information at creating new drugs with desired properties.

The interdisciplinary of research creates additional difficulties in creating new drugs. Currently, there are no universal algorithms and software for predicting the “structure-property” dependence of drug compounds that can take into account the needs of specialists in this field.

In this regard, the development of a modern Smart-system based on the promising bio-inspired approach of artificial immune systems for predicting the structure-property dependence of drug compounds is relevant.

The aim of this work is to develop a multi-agent Smart-system for predicting the “structure-property” dependence of drug compounds using the ontological approach and modified algorithms of artificial immune systems using the example of drug compounds of the sulfonamide group. The proposed system makes it possible to increase the accuracy of prediction models of the “structure-property” dependence, to reduce the time and financial costs for obtaining candidate drug compounds.

**Methods:**

During the creation of a Smart-system, there are used multi-agent and ontological approaches, which allow to structure input and output data, optimally to distribute computing resources and to coordinate the work of the system. As a promising approach for processing a large amount of chemical information, extracting informative descriptors and for the creation of an optimal data set, as well as further predicting the properties of medicinal compounds, there are considered modified algorithms of artificial immune systems and various algorithms of artificial intelligence.

**Results:**

There was developed an ontological model of a multi-agent Smart-system. There are presented the results of the «structure-property» dependence simulation based on a modified grey wolf optimization algorithm and artificial immune systems. During the simulation, there was used information from the Mol-Instincts sulfonamide descriptor database.

**Conclusion:**

The developed multi-agent Smart-system using ontological models allows visually to present the structure and interrelationships of agents functioning, which greatly facilitates the development of software and reduces time and financial costs during the development of new drugs.

## Background

The rapid development of information technologies and innovative approaches of artificial intelligence create ample opportunities for the development of modern technologies of computer molecular design of medicinal compounds with desired properties [1]. Great successes in recent years have been achieved in the field of predicting the “structure-property/activity” dependence (Quantitative Structure-Activity Relationship, QSAR) of organic compounds at the creation of new drugs. Scientific publications on QSAR prediction appeared in the mid-60s. One of the first researchers in this field is K. Ganich and T. Fujita [2]. The process of creating new drugs is complex, consisting of many stages and is associated with large financial as well as time costs. We obtained large theoretical results on the development of actual intellectual methods in this field of research. The book [3] provides an interdisciplinary review of recent achievements in the QSAR methodology using artificial intelligence algorithms. Various applications were considered, including traditional ones (in chemistry, pharmaceuticals, ecology, and agricultural science) and non-traditional ones, such as in Food Science and Nanoscience.

A promising trend in the development of QSAR is the use of bio-inspired intellectual methods. Researches are actively developing on the basis of artificial neural networks (NN), evolutionary and genetic algorithms (GA), algorithms of swarm intelligence (SI), artificial immune systems and many others. For example, in work [4], there are considered questions regarding the use of neural networks (NN) for the QSAR problem. There are presented the main trends in the development of neural networks in this area, there are analyzed the main advantages and disadvantages of the NN approach. Researches [5] showed that the use of deep neural networks (DNN) is a promising direction and gives a better result than traditional methods. The obtained results were compared with the random forest (RF) method for a diverse set of QSAR data. It is also shown that DNN multitasking models that are trained and predict several QSAR properties are superior to DNNs that have been trained on separate data sets for most tasks. An efficient prediction strategy using multi-tasking DNNs has been developed.

Genetic algorithms (GA) are widely used [6] for quantitative modeling of the «structure- property/activity» dependence. The article discusses the basic principles underlying the GA and provides an overview of recent applications in QSAR with particular emphasis on the use of GA in the choice of characteristics and for the reduction of dimension, as well as for the optimization of models. It is shown that the use of GA allows to obtain accurate and reliable forecasts. In [7], the QSAR problem is solved for the analysis of antimalarial activity of 68 urea derivatives using multiple linear regression (MLR). A suitable set of molecular descriptors (topological, geometric, electrostatic, quantum chemical, etc.) was selected using a genetic algorithm (GA). The results showed a good prognostic ability of the model and the ability to use it for the creation of a similar group of antimalarial compounds. The article [8] discusses the development of a linear quantitative model of the structure-activity ratio in order to predict the activity of inhibiting the ribosomal S6 kinase (RSK) of some new compounds. Multiple linear regression (MLR) was used as a tool for selecting variables in combination with GA. The results showed that the GA-MLR model is applicable for the development of new RSK inhibitors. In studies [9], new models of the structure-property quantitative ratio are presented for predicting the flash point of binary liquid mixtures. More than 600 experimental flash points were used for 60 binary mixes. There is considered a model based on the use of a genetic algorithm and multiple linear regression (GA-MLR). The results show a good predictive ability of the model and the possibility of using mix descriptors.

There were published many papers on the application of modified algorithms of swarm intelligence for solving optimization problems. The article [10] is devoted to the actual problem of the selection of informative descriptors based on algorithms of swarm intelligence for various applications. The analysis shows that more than 60% of such problems are solved in biomedicine. In work [11], a hybrid swarm algorithm is considered for a diagnosis in various diseases and for minimizing the error of an incorrect diagnosis. There was developed a hybrid algorithm based on the ant colony algorithm and the support vector machine. Testing was performed using five basic sets of medical data on various diseases from the UCI repository (UCI Machine Learning Repository) and showed a good result. Researches [12] are devoted to QSAR modeling questions for prediction of λmax of dyes based on of 9,10-anthraquinone derivatives. Using the HyperChem and Dragon programs, there were calculated 1514 descriptors. The main problem of QSAR is the high dimension of the descriptor space; therefore, the choice of descriptor is the most important step. The best descriptors are selected using the metaheuristic algorithm of ant colony optimization (ACO). Informative descriptors were used to develop a model using multiple linear regression. Simulations have shown that using ACO gives a good result.

In article [13], there is used the structure-activity quantitative ratio to predict the activity of one group of newly synthesized halogenated pyrimidine derivatives as inhibitors of human dihydroorotat dehydrogenase using a modified bee algorithm. The molecular structures of the halogenated pyrimidine derivatives were obtained in HyperChem, and the molecular descriptors were calculated using the DRAGON software. The most efficient descriptors for 32 halogenated pyrimidine derivatives were selected using a bee algorithm. The correlation coefficients for training and test cases were obtained as 0.9596 and 0.9185, respectively. The simulation results showed that the bee algorithm has good characteristics for the selection of variables in the QSAR researches and gives a better result compared to the genetic algorithm. In studies [14], there is considered the use of a bee algorithm (BA) as a method for the selection of descriptors for studying the retention of pesticides in biopartitioning micellar chromatography.

Nowadays, information systems for prediction and diagnostics are actively developed using various modified algorithms of artificial immune systems. Studies [15] are devoted for the prediction of a protein structure based on an algorithm of artificial immune systems using quantum clonal selection algorithm (QCSA). The article [16] proposes the AIRS-GA hybrid approach based on the Artificial Immune Recognition System (AIRS) and the deterministic version of the genetic algorithm (GA). The experiments were conducted on real data sets obtained in the United States. Tests have shown that the modified AIRS-GA algorithm is superior to the original AIRS algorithm in terms of classification accuracy and in time. The article [17] discusses the hybrid clonal selection algorithm MSHCSA with modified combinatorial recombination and adaptive mutation for solving numerical optimization problems. This algorithm allows to solve complex optimization problems such as: poor ability to search, premature convergence. The proposed algorithm has been tested and a comparative analysis was carried out with modern evolutionary algorithms. Experimental results show that this algorithm is competitive.

Today, multi-agent technologies are actively developing for the creation of information systems for various purposes based on the implementation of modern intelligent algorithms. For example, in article [18] the multi-agent AIS model is considered for recognition of programs infected by viruses. A multi-agent system (MAS) consists of a set of autonomous agents operating in a software environment and allows recognition with minimal computational resources. The advantages of using MAS are: flexibility of operation, high self-organization, scalability, the ability to interact between agents, the optimal distribution of computational resources, the choice of the strategy of agents behavior, taking into account the experience of interaction with the software environment and multi-functionality. Multi-agent systems are a promising direction in medicine and pharmacology. Article [19] is devoted to the problem of the development of personalized medicine for the elderly using modern multi-agent information technologies. Using 3D sensors that are worn by older people and the corresponding intelligent software environment creates tremendous opportunities for monitoring functionality, for the prediction of the condition and timely adjusting of patients’ health.

Due to the fact that the researches on computer molecular design of drugs with desired properties are interdisciplinary in nature and affect various scientific areas such as: organic chemistry, molecular biology, bioinformatics, chemometrics, computer modeling, information technology and artificial intelligence, then it is actual the development and the use of ontological models [20, 21], allowing to systematize the used intelligent algorithms and to structure the data for application of various methods for solving this problem. The article [22] is devoted to the use of ontologies in data management for artificial intelligence. Researches [23] consider the use of ontologies in the health care system for monitoring patients and detecting abnormal situations. The work [24] is devoted to the development of an ontology for a database of immune epitopes. In article [25], there was developed a model of an artificial immune system based on ontology in the Protégé editor.

A review of current publications on this topic has shown that the use of various modified algorithms of artificial immune systems and other modern approaches of artificial intelligence, as well as multi-agent technologies and the ontological approach is a relevant and promising direction in processing multidimensional data and for solving prediction problems aimed at reducing financial and time costs during the selection procedure of candidates of new chemical compounds with given pharmacological properties for further research.

The following structure of the article is proposed: the second section is devoted to the formulation of the research problem and to the necessary requirements for the creation of a multi-agent Smart-system for conducting scientific research on QSAR prediction. The third section discusses solution methods and algorithms, provides a block diagram of a multi-agent Smart-system of prediction, and also presents an ontological model created in the ontology editor Protégé. The description of the molecular structure of sulfanilamide group compounds with different pharmacological activity in the form of descriptors is given. The fourth section is devoted to the results of QSAR modeling for antiseptic sulfonamide drug compounds based on a modified algorithm using the grey wolf algorithm (GWO) and artificial immune systems (AIS). The fifth section provides the conclusion and the list of references.

## Methods

### Problem statement

The problem statement is formulated as follows: it is necessary to develop an ontological model of a multi-agent Smart-system for conducting scientific researches in order to predict the «structure-property/activity» dependence of medicinal compounds based on modified algorithms of artificial immune systems using the example of computer molecular design of new pharmaceutical antiseptic drugs of sulfanilamide group with a given biological activity.

There were developed necessary requirements for a multi-agent Smart-system for conducting scientific researches on QSAR prediction, which should be taken into account at processing multi-dimensional structural chemical information:

- combining advanced methods in biomedicine and pharmacology, computing, the latest achievements of artificial intelligence and the use of ontological approach;
- the use of modern databases of chemical information;
- the ability to process large volumes of structural chemical information;
- convenient and understandable user interface;
- modular structure and ability to expand the system;
- rather high speed of information processing due to the use of parallel computing technologies;
- the ability to connect to the modern application packages and libraries for processing and visualization of big data;
- application of cloud technologies;
- ease of working with the system without long training.

### Dataset

Researches were conducted on the example of a database of medicinal compounds of sulfonamides.

One of the main tasks in computer molecular design of medicinal compounds is the description of the molecular structure of the chemical compounds under consideration in the form of descriptors that characterize their specific properties. There is a classification of different levels of descriptors [26]: the elementary level, the structural formula, the electronic structure, the molecular form, and the descriptors of intermolecular interactions.

The studies used information on sulfonamides from the database of Mol-Instincts chemical substances, which is the most extensive and describes more than 2.85 million chemical substances, and also contains about 10 billion chemical data sets. There is developed database of sulfonamides of different duration of action: short - less than 10 h (streptocid, sulfadimidine, etc.), medium - 10-24 h (sulfadiazine, sulfamethoxazole, etc.), long-lasting - 24-48 h (sulfadimethoxine, sulfamonomethoxin, etc.). Table 1 presents a fragment of the base.

**Table 1:** Sulfonamides. A fragment of the sulfonamide descriptors database

ID acc. Mol-Instincts base	Chemical formula	SMILES specification (Simplified Molecular Input Line Entry Specification)	2D structure
0001-mip8	C12H14N4O4S	COc2cc(NS(=O)(=O)c1ccc(N)cc1)nc(OC)n2	
0001-m9es	C12H14N4O2S	Cc2cc(C)nc(NS(=O)(=O)c1ccc(N)cc1)n2	
0001-ixe4	C11H13N3O3S	Cc2noc(NS(=O)(=O)c1ccc(N)cc1)c2C	
0001-ney3	C9H10N4O2S2	Cc2nnc(NS(=O)(=O)c1ccc(N)cc1)s2	
0000-jjsq	C10H11N3O3S	Cc2cc(NS(=O)(=O)c1ccc(N)cc1)no2	
…	...	...	...

Each substance is described by 2005 descriptors, such as: structural descriptors (the amount of atoms, the relative amount of atoms C, H, O, N, S atoms; the number of single links, etc.); topological descriptors (simple Narumi topological index; branch index; Schultz molecular topological index, etc.); descriptors describing distances and trajectory calculation (molecular distance; total distance, etc.); descriptors characterizing the binding index (valence binding index; modified Randic binding index, etc.); information indices (information about the size of the molecule; the general index of atomic composition information; average content of information about the distance; index of complexity of the graph vertices; information about the amount of links; content of structural information, etc.), etc. The developed database consists of a total of 30,075 data instances. A fragment of the sulfonamide descriptor database is shown in Table 2.

**Table 2:** Sulfonamides. A fragment of the sulfonamide descriptor database

№	Descriptor	Sulfadiazine	Sulfadimidine	Sulfafurazole	...	Sulfalen	Sulfaperin
D1	Number of atoms	27.000	33.00	31.000	...	31.000	30.000
D2	Relative number of C atoms	0.3704	0.363	0.354	...	0.3548	0.366
D3	Relative number of H atoms	0.3704	0.424	0.419	...	0.3871	0.400
D4	Relative number of O atoms	0.0741	0.0606	0.096	...	0.096	0.066
D5	Relative number of N atoms	0.148	0.1212	0.096	...	0.129	0.133
D6	Relative number of S atoms	0.037	0.0303	0.032	...	0.032	0.0333
D7	Number of single bonds	16.000	24.000	26.000	...	26.000	22.000
D8	Relative number of single bonds	0.571	0.7059	0.8125	...	0.8125	0.709
D9	Relative number of double bonds	0.000	0.00	0.00	...	0.00	0.00
D10	Relative number of triple bonds	0.000	0.00	0.00	...	0.00	0.00
D11	Number of aromatic bonds	12.000	10.0000	6.000	...	6.000	9.000
D12	Relative number of aromatic bonds	0.428	0.294	0.187	...	0.1875	0.290
D13	Relative number of rings	0.0741	0.0606	0.0645	...	0.064	0.066
D14	Relative number of benzene rings	0.00	0.0303	0.00	...	0.00	0.033
D15	Molecular weight	250.275	278.328	267.30	...	280.30	264.301
D16	Relative molecular weight	9.269	8.434	8.622	...	9.042	8.8101
D17	Gravitation index (all bonds)	1980.80	1916.90	1874.9	...	1960.3	1843.00
D18	Gravitation index (all pairs)	3794.10	4085.10	3758.2	...	4159.3	3711.20
D19	Cubic root of Gravitation index (all bonds)	12.558	12.422	12.330	...	12.515	12.260
D20	Cubic root of Gravitation index (all pairs)	15.596	15.985	15.547	...	16.082	15.482
D21	average molecular weight	9.270	8.440	8.620	...	9.0400	8.8100
D22	sum of atomic van der Waals volumes	17.880	21.080	19.590	...	19.990	19.480
D23	sum of atomic Sanderson electronegativities	27.790	33.560	31.780	...	32.000	30.670
D24	sum of atomic polarizabilities	18.860	22.390	20.840	...	21.080	20.630
D25	sum of Kier-Hall electrotopological states	46.580	46.170	43.500	...	49.830	41.830
D26	mean atomic van der Waals volume	0.660	0.6400	0.63000	...	0.640	0.650
D27	mean atomic Sanderson electronegativity	1.030	1.020	1.0300	...	1.030	1.020
D28	mean atomic polarizability	0.700	0.6800	0.67000	...	0.680	0.690
D29	mean electrotopological state	2.740	2.4300	2.4200	...	2.620	2.320
D30	number of non-H atoms	17.000	19.000	18.000	...	19.000	18.000
…	...	...	...	...	...	...	…
D2005	HACA-2/sqrt(TMSA) [Zefirov]	0.062	0.047	0.031	...	0.034	0.0316

In order to create a stable QSAR model and to obtain a qualitative forecast, it is very important to create an optimal set of sulfonamide descriptors, which maximally stores information about the molecular structure of the compound with the minimum amount of descriptors. Therefore, it is important to apply various optimization modified algorithms of artificial intelligence to solve this problem.

## Methods and algorithms of research

Since there are no universal prediction algorithms, then at the creation of this application there is used a multi-algorithm approach, which involves the use of various intelligent algorithms and their modifications to solve effectively the problem.

Currently, this approach is widely used to solve the classification problem. For example, the method of multi-algorithmic classification was considered in [27]. It describes a model for calculating estimates, based on a system of logical laws for solving the classification problem with a teacher. In studies [28], a comparative analysis of multi-algorithmic and multimodal approaches in an application for biometric systems is presented. There are given the examples of a multi-algorithmic approach based on the algorithms of principal component analysis, fisher linear discriminant, independent component analysis. In [29], there is presented a multimodal biometric system in which a multi-algorithmic approach is used to reduce the data set. Researches [30] are devoted to the application of a multi-algorithm approach for recognizing a person’s electrocardiogram. The multi-algorithmic approach includes a combination of the autocorrelation method and the wavelet transform, which work in parallel. In the current work, a multi-algorithmic approach is understood as the following definition given below.

Remark 1. A multi-algorithmic approach is an approach in which several intelligent algorithms are used at once to solve the problem of selecting informative descriptors of drug compounds and for predicting the «structure-property» dependence of drug compounds (for example, to reduce data: gray wolf optimization method, flower pollination algorithm, random forest and algorithms based on artificial immune systems for solving the problem of prediction), which are calculated simultaneously. Based on the results of the calculations, a combination of algorithms is selected that gives the best predictive result [31].

Therefore, the multi-algorithm approach is an effective tool for increasing the prognostic ability of the model and can be successfully used in pharmacology to obtain new drug compounds.

### Artificial immune system approach

The central nervous system and the immune system are complex highly organized systems that participate in the metabolic processes of the whole organism and have common features in the functioning mechanisms [32]. However, although the properties of individual neurons are better studied than the properties of any other cells, the brain as a whole remains the most mysterious organ of the body [33]. Meanwhile, the latest molecular biology methods demonstrate the high efficiency of the information processing realized by proteins, as well as the unity of the principles of their functioning in all regulatory systems of the body (hormonal, immune, nervous).

The natural neural system and the artificial immune system of vertebrates possess the capabilities of “intellectual” information processing [34]. These systems have memory, the ability to learn, recognize and the ability to make decisions. Neural networks have become widespread as a computational model, but the computational capabilities of artificial immune systems have been evaluated relatively recently.

Currently, there are described various specific mechanisms of immunity functioning. For example, the immune network modeling algorithm [35] is based on molecular recognition between formal peptides, etc. There are several main directions of AIS based on clonal selection [36], immune network modeling (INM) and negative selection (NS) [37]. There are developed many modified algorithms for these areas. Of great interest is the comparison of QSAR prediction results based on modified AIS algorithms using different approaches to create an optimal set of descriptors.

### Artificial immune recognition systems

In order to solve the problem of pattern recognition, the Artificial Immune Recognition System (AIRS) algorithm has established itself. Let consider the mechanism of AIRS functioning. The operation of the AIRS algorithm can be divided into 4 main stages: Stage 1 - data initialization and normalization; Stage 2 - identification of memory cells and generation of ARB (Artificial Recognition Ball); Stage 3 - the struggle for resources at creating a candidate memory cell; Stage 4 - training the algorithm by introducing a potential candidate memory cell into established memory cells.

Throughout life, a large number of pathogens [38] enter the human body. In order to fight them, the human immune system also uses lymphocytes, which have receptor molecules on their surface that recognize pathogenic microorganisms. Receptors contain certain parts that are able to attach to a foreign molecule (antigen). The AIRS algorithm uses the antigen/antibody binding mechanism to describe the training process in which training data (antigens) are compared and potential solutions *B* - are cells. The authors J. Timmis and M. Neal (2001) introduced the concept of ARB (Artificial Recognition Ball).

As soon as the proximity between the *B*- cell and the antigen is established, the *B*-cell is transformed into a plasma cell and the mechanism of clonal expansion begins. During clonal expansion, the *B*- cell undergoes rapid cloning. This response is specific for antigen. These clones then pass through matrix maturation (affinity maturation), i.e. the process of increasing affinity of antibodies to a sensitive antigen as the immune response develops. Some clones undergo somatic hypermutation, after which this cell can become a memory cell. Memory cells provide a quick response to the same similar antigen in case of re-infection. A similar mechanism is called a secondary immune response. In AIRS, the idea of clonal expansion and matrix maturation is used to stimulate the generation of potential memory cells, which are then used for classification. In the AIRS algorithm, ARBs compete for survival based on a system with limited resources. Non-competitive ARBs are removed from the system. In the AIRS algorithm, the *B* - cell population is constantly changing due to cell proliferation and death. The AIRS method is promising for solving the problem of predicting the “structure-property/activity” dependence of drug compounds.

### Clonal selection

One of the common and promising algorithms is the algorithm of artificial immune systems based on clonal selection. Currently, there are many modifications of this algorithm, such as: CLONALG, CLONALG1, CLONALG2, CLONCLAS and Adaptive Clonal Selection [39].

The clonal selection algorithm is based on the theory of the scientist Burnet F.M [40]. The theory of clonal selection is a fundamental principle of modern immunology [41] and describes the behavior and capabilities of antibodies in the acquired immune system. After the lymphocyte is selected and binds to the antigenic determinant (a small portion of the antigen molecule), forming a spatial configuration, which is the point of connection of the antibody molecule, the cell begins to reproduce rapidly. Thousands of cell copies are created, which are transformed into plasma cells and memory cells. Plasma cells produce a huge number of antibody molecules and have a short lifespan. The memory cells, on the contrary, live for a long time.

According to the theory of clonal selection, during the process of reproduction and copying, the cell undergoes small copying errors (somatic hypermutation). Somatic hypermutation results in changes in the genome that change the shape of the expressed receptors. As a result, there is changed the ability to recognize antibodies associated with the surface of lymphocyte cells and antibodies that produce plasma cells.

Therefore, the enlarged clonal selection algorithm includes the following mechanisms: clonal selection, clonal expansion, and and affinity maturation via somatic hypermutation.

### Immune network algorithm (AIS)

A promising approach is artificial immune systems based on the principles of molecular recognition [42]. In this case, the basic element is a formal peptide. A formal peptide is understood as a mathematical abstraction of the free energy of a protein molecule from its spatial form. This is a relatively new direction in artificial intelligence, using which a number of applications have been developed [31, 43]. The main problems that arise during immune network modeling are: the choice of the structure of the immune network; reduction of training time; solving the problem of informative features selection; increase the reliability of the prediction and parallelization of computational algorithms.

Remark 2. Under the optimal structure of the immune network there is meant a network created on the basis of the weight coefficients of the selected informative descriptors, which most fully describe the chemical compound under consideration. The criterion is the maximum storage of information with a minimum number of descriptors.

Since in the AIS approach, a binding network is understood as any sequence of binding of formal peptides, in order to create an immune network model, it is necessary to create formal peptides (time series) that will serve as standards (connection with desired properties). Formal peptides consist of informative descriptors characterizing the drug compound. The time series are folded in a certain way into the matrix of standards, after the singular decomposition of these matrices, the right and left singular vectors of the standard matrices are obtained, then many matrices are formed that are considered as patterns, the binding energy between the formal peptides is calculated. Using singular decompositions of the initial matrices, the binding energies are determined. The problem of pattern recognition is being solved. The minimum value of the binding energy determines the class to which this pattern belongs to. Next, the energy errors are estimated by homologs [43]. Then there is conducted the prognosis and selection of drug candidate compounds.

### Naïve Bayes algorithm

The naive Bayesian algorithm is based on the Bayesian theorem and is one of the simple, but at the same time very efficient classification algorithms. Bayes theorem allows to calculate the posterior [44]:

1 $$ P\left(c|x\right)=\frac{P\left(x|c\right)P(c)}{P(x)} $$

The posterior probability of this class *c* for a given value of the feature *x* is denoted as *P*(*c*|*x*). The a priori probability of this class is *P*(*с*). The parameter *P*(*x*|*c*) indicates the probability of a given feature value in this class. The a priori probability of a given feature value is defined as *P*(*х*).

The naive Bayesian classification algorithm has the following advantages: speed, the need for a small amount of training sample, works better with categorical features than with continuous ones. However, the values of the predicted probabilities are not always accurate enough, and the assumption of independence of features is also a disadvantage, since completely independent symptoms are rare.

### Logistic regression

Logistic regression is a tool for solving the problems of regression and classification. It is successfully used for creating models in medicine and for conducting clinical studies, as well as in the field of QSAR modeling [45]. The algorithm analyzes the relationship between several independent variables (regressors or predictors) and the dependent variable. There is a binary logistic regression if the dependent variable is binary. Logistic regression allows to evaluate the probability whether an event occurs for a particular object.

The advantages of this algorithm are [46]: the ability to evaluate posterior probability and risks, as well as the relative ease of implementation. The disadvantages are: the need for data standardization, screening out outliers and the selection of features to improve convergence.

### Decision tree

**Fig. 1:**
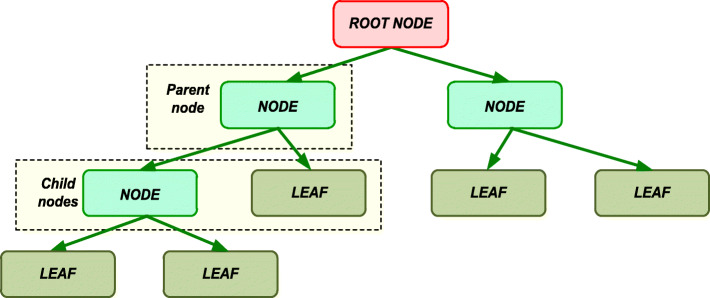
Decision tree structure creation

The decision trees algorithm is a machine learning algorithm and is used to solve the classification and regression problems [47]. By analogy with wildlife, the algorithm consists of elements of “leaves” and “branches”. The “branches” contain the values of the attributes on which the objective function depends; the value of the objective function is recorded in the “leaves”. Also, one of the elements of the algorithm are the “nodes” in which the decision rules are located. The Fig. 1 gives a general view of the decision tree structure.

The advantages of this algorithm are: ease of implementation, there is no need for data preprocessing, it allows evaluating the model using statistical tests and the ability to process large amounts of data. The disadvantages of the method include: the problem of obtaining the optimal decision tree, retraining, the risk of obtaining too large decision tree, etc.

### Random Forest

At present, the Random Forest algorithm proposed by Breiman [48] for solving the problems of classification, regression, clustering, and selection of informative features, is widely used. The method has applications in various fields, including successfully applied in the field of QSAR modeling [49]. The algorithm consists of a combination of decision trees, which are the numerical parameter of the method. Each tree depends on the value of a random vector of independent sample with the same distribution as for all trees in the forest [48]. The error of generalizing the forest of decision trees depends on the size of individual trees in the forest and the correlation between them. Thus, due to the ensemble of decision trees that are themselves less efficient, the Random Forest algorithm is a stronger algorithm.

The advantages of the algorithm are the ability to process data with a large number of features and classes, insensitivity to scaling, and high parallelism. The disadvantages include the large size of the models, which leads to an increase in the requirements for the computer memory and simulation time.

### Support vector machine

**Fig. 2:**
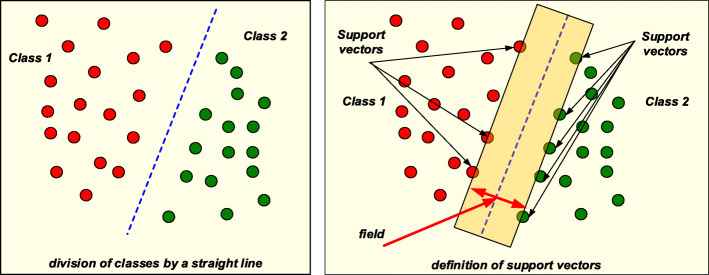
Support vector method

The support vector method (SVM) is mainly used to solve the classification problem [50] and is based on the concept of hyperplanes [51]. The boundaries of decision making are determined using decision planes. A plane divides features into classes. The Fig. 2 shows an example in which objects belonging to two different classes are involved. The aim of the SVM method is to find a plane separating two sets of objects. The algorithm finds features that lie on the boundary of classes. These sets are called support vectors. The classification result is considered good if the area between the boundaries is empty.

The advantages of the method include the ability to work with a small data set, ease of implementation, minimal classification error, and the ability to work with a real data set. The disadvantage of this method is that during the solution of the classification problem, not all data is used, but a small part of it, which is located at the boundaries.

### Multi-agent Smart-system for QSAR prediction

Actual is the use of multi-agent systems in order to solve the problem and to create a QSAR prediction system. A set of agents is created (database agent, ontology agent, descriptor optimization agents, AIS agents and a decision agent) with various functions for implementation of the used intelligent algorithms. All agents have a description of their range of tasks and can interact in a software environment with each other. A library of algorithms is created for the implementation of all approaches in the Smart-system operation. The multi-agent approach provides multi-functionality of the system, resistance to system errors, as well as optimization of computing resources.

**Fig. 3:**
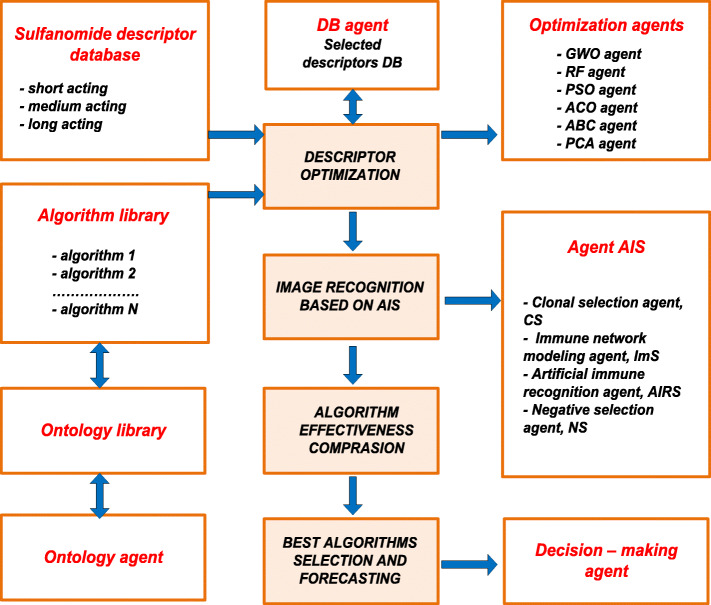
Smart-system. A block diagram of the multi-agent Smart-system operation

There was developed a block diagram of the multi-agent Smart-system for QSAR prediction (Fig. 3). Below is the algorithm of the multi-agent Smart-system operation.

### Algorithm 1

Step 1. Connection of the database of structural chemical information descriptors characterizing the medicinal compounds under consideration. As an example, there are considered sulfonamides with different duration of pharmacological activity (short, medium and long).

Step 2. Selection of data optimization method: grey wolf algorithm, random forest algorithm, particle swarm algorithm (PSO), ant colony optimization algorithm, bee colony algorithm, principal component analysis method (PCA) for reducing uninformative descriptors and for the creation an optimal set of descriptors.

Step 3. Creation of an optimal immune model based on a selected set of descriptors. The redundancy and low information content of descriptors reduces the quality of the forecast, therefore, selecting new chemical compounds for drug candidates, it is important to take into account the descriptive information content.

Step 4. Selection of an artificial immune system algorithm: based on clonal selection (CS), immune network algorithm (INS), artificial immune recognition system algorithm (AIRS), negative selection algorithm in order to solve the image recognition problem and for the prediction.

Step 5. Training of AIS on standards, composed by experts from descriptors of medicinal compounds with precisely known properties (short, medium, long-lasting and extra-long action).

Step 7. Image recognition based on the selected AIS algorithm.

Step 8. Predicting QSAR of chemical compounds.

Step 9. Comparison of the effectiveness of algorithms.

Step 10. Selection of the best algorithms and a forecast based on them. Selection of candidates for new chemical compounds with desired properties for further researches.

### Ontological model of multi-agent smart-system

In the ontology editor Protégé [52] there is developed an ontological model of the multi-agent Smart-system for OM_MSR_ prediction:

2 $$ {\mathrm{OM}}_{\mathrm{MSR}}=<{\mathrm{OM}}_{\mathrm{OD}},{\mathrm{OM}}_{\mathrm{AIS}}> $$

where OM_OD_ – ontological descriptor optimization model, OM_AIS_ - ontological model of artificial immune systems algorithms.

The OM_OD_ ontological model consists of the following tuple:

3 $$ {\mathrm{OM}}_{\mathrm{OD}}=<{\mathrm{OM}}_{\mathrm{GWO}},{\mathrm{OM}}_{\mathrm{RF}},{\mathrm{OM}}_{\mathrm{PSO}},{\mathrm{OM}}_{\mathrm{ACO}},{\mathrm{OM}}_{\mathrm{ABC}},{\mathrm{OM}}_{\mathrm{PCA}}> $$

where OM_GWO_ – ontological model of gray wolves algorithm, OM_RF_ – ontological model of a random forest algorithm, OM_PSO_ – ontological model of particle swarm optimization algorithm, OM_ACO_ – ontological model of ant colony algorithm, OM_ABC_ - ontological model of a bee colony algorithm, OM_PCA_ – ontological model of the principal component method.

The ontological model of image recognition and OM_R_ prediction is as follows:

4 $$ {\mathrm{OM}}_{\mathrm{R}}=<{\mathrm{OM}}_{\mathrm{IsM}},{\mathrm{OM}}_{\mathrm{ClS}},{\mathrm{OM}}_{\mathrm{NgS}},{\mathrm{OM}}_{\mathrm{AIRS}}> $$

where OM_IsM_ – ontological immune network model, OM_ClS_ – ontological model of artificial immune systems based on clonal selection, OM_NgS_ - ontological model of artificial immune systems based on negative selection, OM_AIRS_ – ontological model based on the image recognition algorithm by an artificial immune system.

**Fig. 4:**
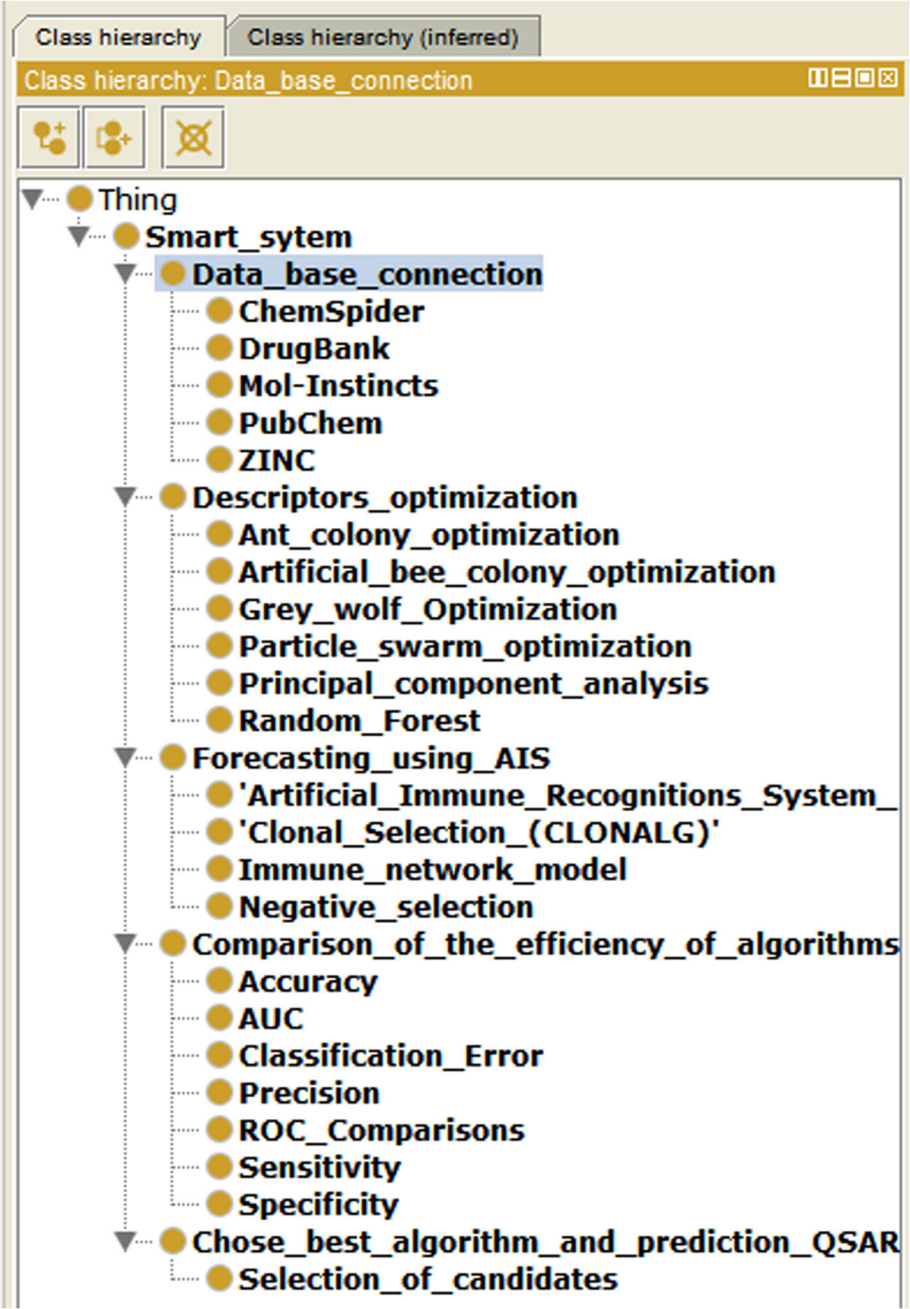
Ontological model. Hierarchical structure of the classes of the ontological model of a multi-agent Smart-system

Figure 4 shows the hierarchical structure of the classes of the developed ontological model of a multi-agent Smart-system created on the basis of tuples (2–4).

**Fig. 5:**
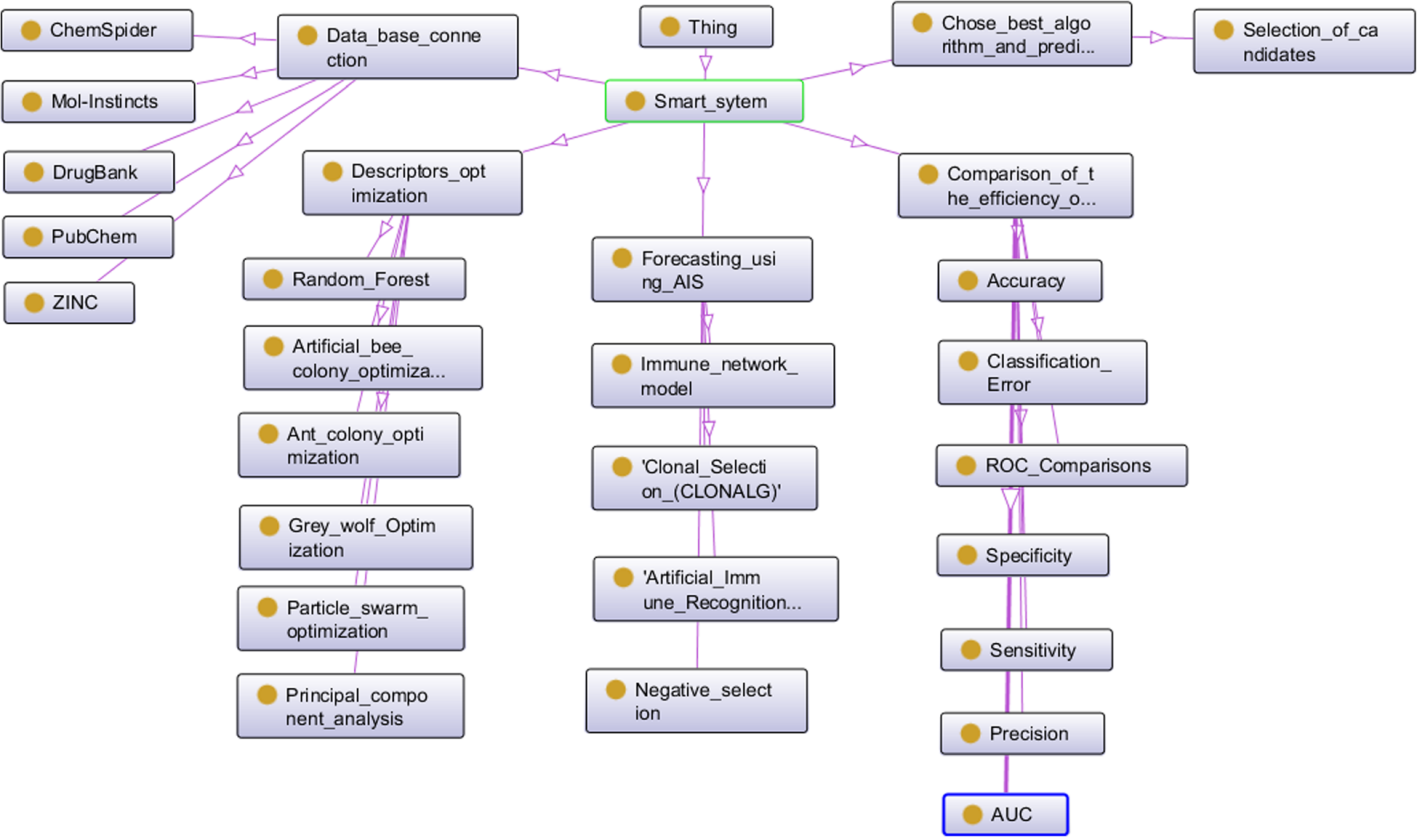
Ontological model. Visualization of the ontological model of a multi-agent Smart-system

Figure 5 shows the visualization of the structure of the ontological model of a multi-agent Smart-system.

The ontological model is used at analyzing the numerous links between agents and takes them into account at software developing. The advantage of a multi-agent Smart-system is the ability to expand with new algorithms and other modules.

### Prediction of QSAR sulfonilamides based on the modified GWO-AIS algorithm using the grey wolf algorithm and artificial immune systems

Let consider an example of QSAR prediction based on drug compounds - sulfonamides. Sulfonamides are antimicrobials with different duration of action. Streptocide is considered as the first synthetic antibacterial agent which is one of the well-known representatives of the sulfonamide group. Preparations of sulfa compounds are widely used against pathogens of infectious and inflammatory processes in medicine and veterinary medicine. However, recently, due to the resistance of microorganisms to drugs of this group, it is important to create new effective drug compounds of sulfonamides with desired pharmacological properties [53].

Let consider an example of predicting the properties of new compounds of the sulfanilamide group using the modified GWO-AIS algorithm. The GWO meta-heuristic algorithm was developed in 2014 by a group of scientists led by S. Mirjalina [54] and is based on the behavior of a pack of grey wolves. The behavior of wolves depends on the occupied hierarchy. Wolves leaders stand out, who occupy the main place in the pack and make decisions, followed by wolves advisers who provide help in decision-making and pass on the decisions to all members of the pack. Then follows a level consisting of several categories: scouts, sentries, elders, hunters, and rangers. Wolves of this level obey the first two levels, but they dominate over the fourth level. The wolves of the last fourth level of the hierarchy obey wolves of all levels. Each of the four wolf hierarchies is assigned its own rank: α, β, δ, and *ω*. Developing the algorithm, the mechanisms of gray wolves behavior were used for searching for the prey, environment, and for attack [55].

The prey environment by wolves is described by the following model:

5 $$ {\displaystyle \begin{array}{l}\overrightarrow{D}=\mid \overrightarrow{C}\cdot {\overrightarrow{X}}_p(t)-\overrightarrow{X}(t)\mid \\ {}\overrightarrow{X}\left(t+1\right)={\overrightarrow{X}}_p(t)-\overrightarrow{A}\overrightarrow{\cdot D}\end{array}} $$

In a model (5) the parameter *t* denotes the current iteration. Vectors-coefficients $\overrightarrow{A},\kern0.5em \overrightarrow{C}$ are calculated according to the following formula:

6 $$ {\displaystyle \begin{array}{c}\begin{array}{l}\overrightarrow{A}=\overrightarrow{2a}\cdot \overrightarrow{r_1}-\overrightarrow{a};\\ {}\overrightarrow{C}=2\cdot \overrightarrow{r_2}\end{array}\\ {}\end{array}} $$

The value $\overrightarrow{a}$ decreases linearly from 2 to 0 in each iteration. Random vectors ${\overrightarrow{r}}_1,{\overrightarrow{r}}_2$ from the interval [0, 1] allow to simulate the movement of wolves. The parameter ${\overrightarrow{X}}_p$ defines the vector position of the victim. The vector position of the wolf is indicated as $\overrightarrow{\mathrm{X}}$. The parameter $\overrightarrow{D}$ is the direction vector from the wolf to the victim.

The hunt process is initiated α, while β and δ can help. In the mathematical model (5) it is shown that α, β and δ represent the best solution regarding the potential location of the prey. The first three best solutions are saved and other agents are required to update their positions according to the position of the best search agents based on equations of the form:

7 $$ {\overrightarrow{D}}_{\alpha }=\mid \overrightarrow{C_1}\operatorname{}\operatorname{}\cdot \overrightarrow{X_{\alpha }}-\overrightarrow{X}\mid, {\overrightarrow{D}}_{\beta }=\mid \overrightarrow{C_2}\cdot \overrightarrow{X_{\beta }}-\operatorname{}\operatorname{}\overrightarrow{X}\mid, {\overrightarrow{D}}_{\delta }=\mid \overrightarrow{C_3}\cdot \overrightarrow{X_{\delta }}-\operatorname{}\operatorname{}\overrightarrow{X}\mid $$

8 $$ \overrightarrow{X_1}=\overrightarrow{X_{\alpha }}-\overrightarrow{A_1}\cdot \left(\overrightarrow{D_{\alpha }}\right),\overrightarrow{X_2}=\overrightarrow{X_{\beta }}-\overrightarrow{A_2}\cdot \left(\overrightarrow{D_{\beta }}\right),\overrightarrow{X_3}=\overrightarrow{X_{\delta }}-\overrightarrow{A_3}\cdot \left(\overrightarrow{D_{\delta }}\right) $$

9 $$ \overrightarrow{X}\left(t+1\right)=\frac{\overrightarrow{X_1}+\overrightarrow{X_2}+\overrightarrow{X_3}}{3} $$

where the vector $\overrightarrow{A}$- is a random value in the range $\left[-2\overrightarrow{\mathrm{a}},\kern0.5em 2\overrightarrow{\mathrm{a}}\right]$, and the value of the parameter a decreases from 0 to 2, according to the current iteration.

For the QSAR prediction there were selected AIS algorithms based on clonal selection and immune network modeling. Below there is a developed modified GWO-AIS algorithm for the creation of an optimal set of descriptors and QSAR sulfonamides prediction.

### Algorithm 2. Modified GWO-AIS Algorithm.

Step 1. Development of a sulfonamide descriptor database.

Step 2. Initialization of the initial data.

Step 3. Classification of sulfonamides according to pharmacological properties.

Step 4. Creation of an optimal set of sulfonamide descriptors to further solution of the problem of image recognition based on AIS.

Step 5. Solution of the problem of AIS image recognition, forecast and decision making [43, 56].

Step 6. The selection of candidates for medicinal compounds of the sulfanilamide group with given pharmacological properties.

### Simulation results

**Fig. 6:**
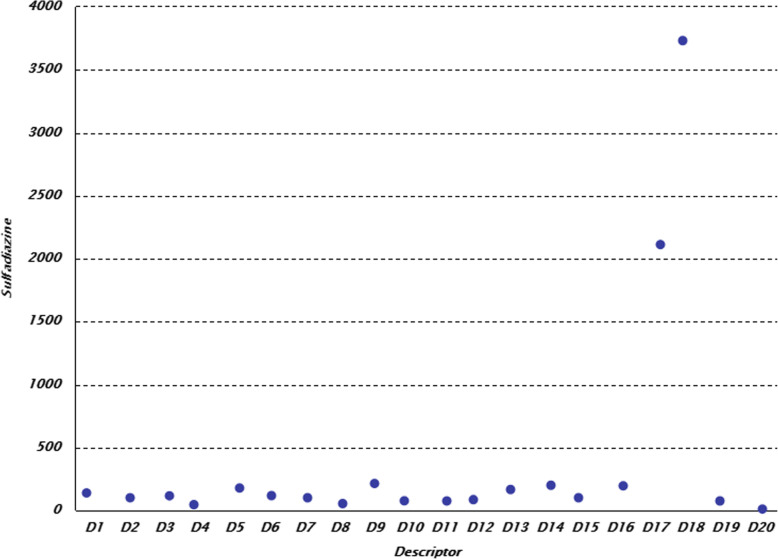
Sulfadiazine. Visualization of a fragment of a descriptors database describing a sulfadiazine substance in the Rapid Miner environment

Let consider the operation of the modified GWO-AIS algorithm. The simulation is based on the Rapid Miner software. Figure 6 shows a fragment of visualization of the sulfadiazine descriptor database.

**Fig. 7:**
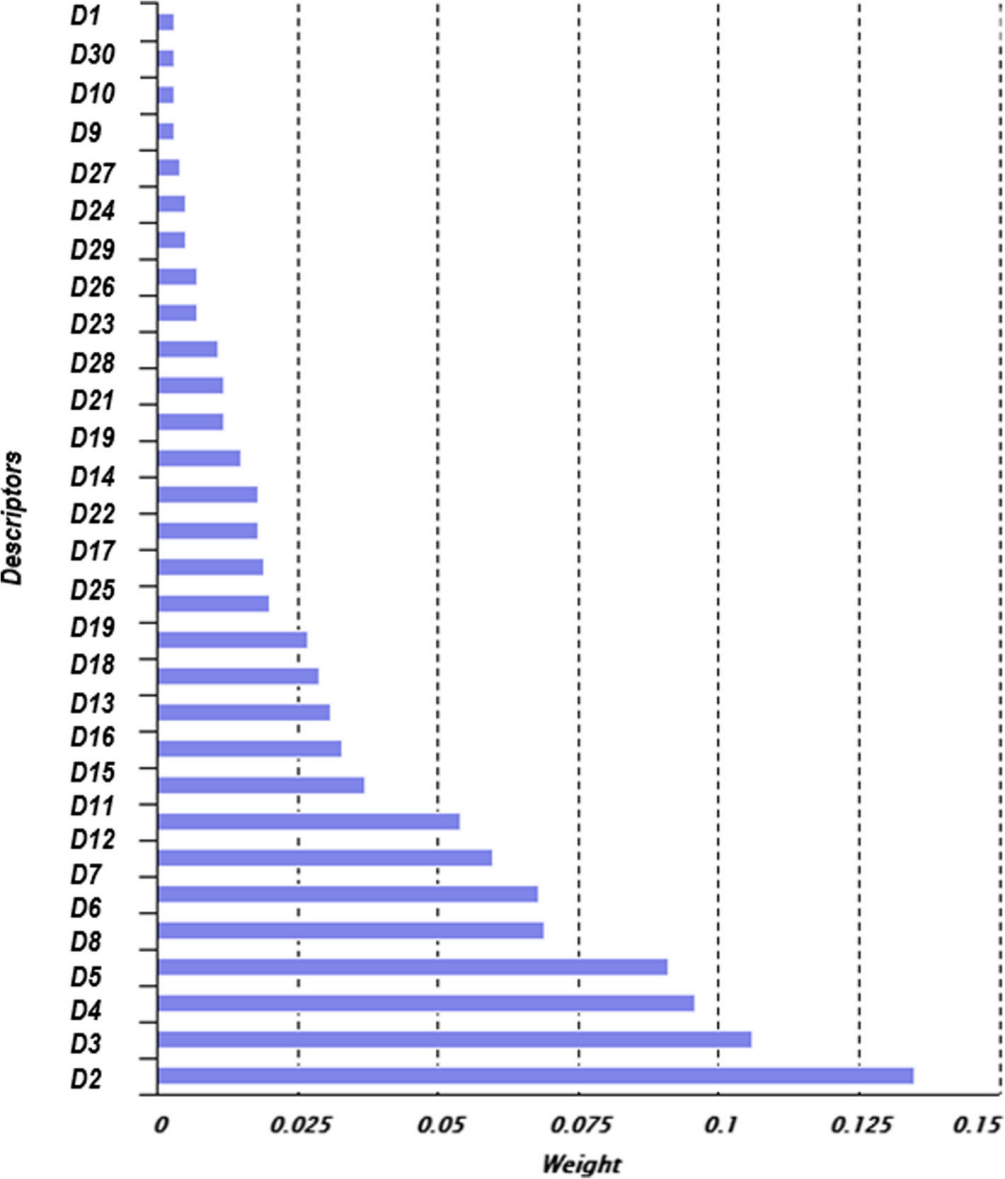
Data reduction. An example of the selection of informative descriptors of chemical compounds of sulfonamides based on the method of grey wolves optimization

Figure 7 shows an example of extracting informative descriptors based on GWO. Descriptors that have the largest value of *weight* parameter are the most informative. The remaining descriptors are the subject for reduction.

After the reduction of non-informative descriptors, the dimension of the DB of sulfonamides is R = 15 × 200, 3000 data attributes.

**Fig. 8:**
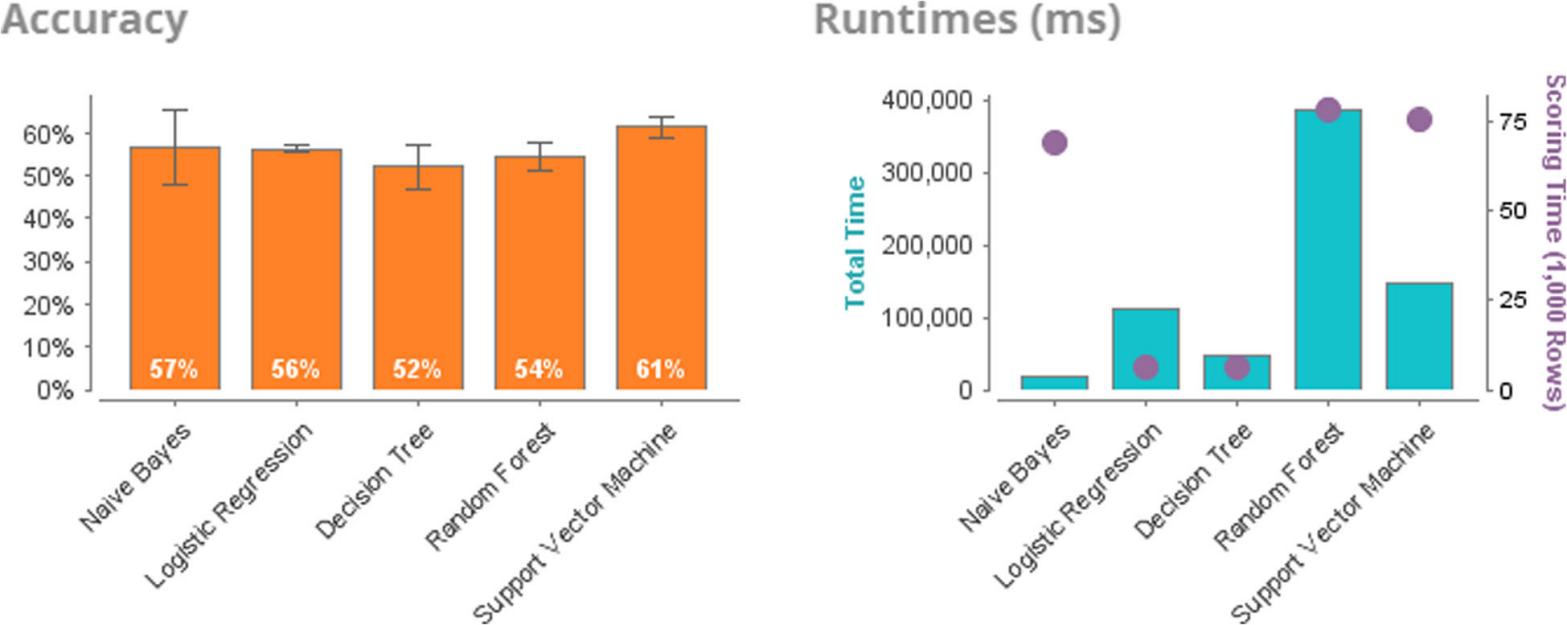
Accuracy. Efficiency of solving the sulfonamides classification problem with different duration of action based on various algorithms of artificial intelligence

To substantiate the effectiveness of the informative descriptors selection based on the GWO, we compare the results of solving the classification problem based on the following image recognition algorithms: Naïve Bayes, Logistic Regression, Decision Tree, Random Forest, Support Vector Machine. Figure 8 shows the results of the analysis of the database without preliminary data processing. Prediction accuracy is low and ranges from 57 to 61%.

Table 3 presents a comparative analysis of the classification algorithms under consideration by the following indicators: accuracy (%), classification error, AUC (Area Under Receiver Operating Characteristic Curve), Precision, Sensitivity, Specificity, Total time, Training Time, Scoring time.

**Table 3:** Sulfanilamides. Comparative analysis of the efficiency of solving the problem of image recognition of the sulfonamide descriptor database

Performance	Naive Bayes	Logistic Regression	Decision Tree	Random Forest	Support Vector Machine
Accuracy	56.9%	56.4%	52.2%	54.4%	61.4%
Classification Error	43.1%	43.6%	47.8%	45.6%	38.6%
AUC	0.586	0.574	0.539	0.552	0.627
Precision	55.5%	56.4%	51.7%	53.3%	61.0%
Sensitivity	59.6%	58.9%	94.0%	82.9%	70.8%
Specificity	53.2%	54.8%	8.8%	24.8%	53.1%
Total Time	17 s	1 min 49 s	46 s	6 min 27 s	2 min 26 s
Training time	2 ms	59 ms	21 ms	241 ms	50 ms
Scoring time	69 ms	6 ms	6 ms	78 ms	75 ms

**Fig. 9:**
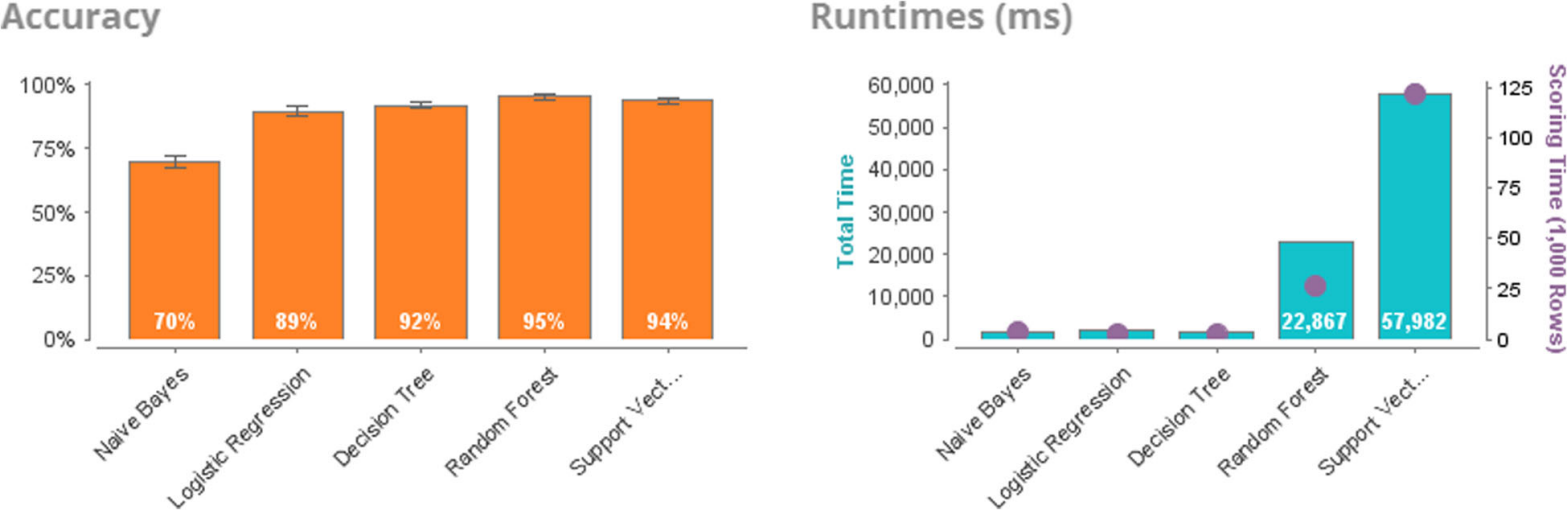
Accuracy. Efficiency of solving the classification problem of sulfonamides with different duration of action based on various algorithms of artificial intelligence after the reduction of non-informative descriptors based on GWO

Next, let consider the prediction results based on the optimal sulfonamide descriptors database, compiled after the reduction of non-informative features using GWO (Fig. 9).

Table 4 presents a comparative analysis of the processing of the optimal database of sulfonamide descriptors. According to the research results, it is clear that the prediction accuracy for each algorithm has increased significantly and the percentage of accuracy is from 69.6 to 93.7%.

**Table 4:** Sulfanilamides. Comparative analysis of the efficiency of solving the problem of image recognition of the optimal database of sulfonamide descriptors

Performance	Naive Bayes	Logistic Regression	Decision Tree	Random Forest	Support Vector Machine
Accuracy	69.6%	89.4%	91.8%	95.5%	93.7%
Classification Error	30.4%	10.6%	8.2%	4.5%	6.3%
AUC	0.494	0.955	0.918	0.983	0.972
Precision	62.2%	84.4%	94.5%	95.6%	93.6%
Sensitivity	99%	95.6%	88.7%	95.6%	93.6%
Specificity	40.7%	83.4%	94.9%	95.4%	93.8%
Total Time	2 s	2 s	2 s	23 s	58 s
Training time	2 ms	189 ms	7 ms	157 ms	94 ms
Scoring time	4 ms	2 ms	2 ms	26 ms	122 ms

**Fig. 10:**
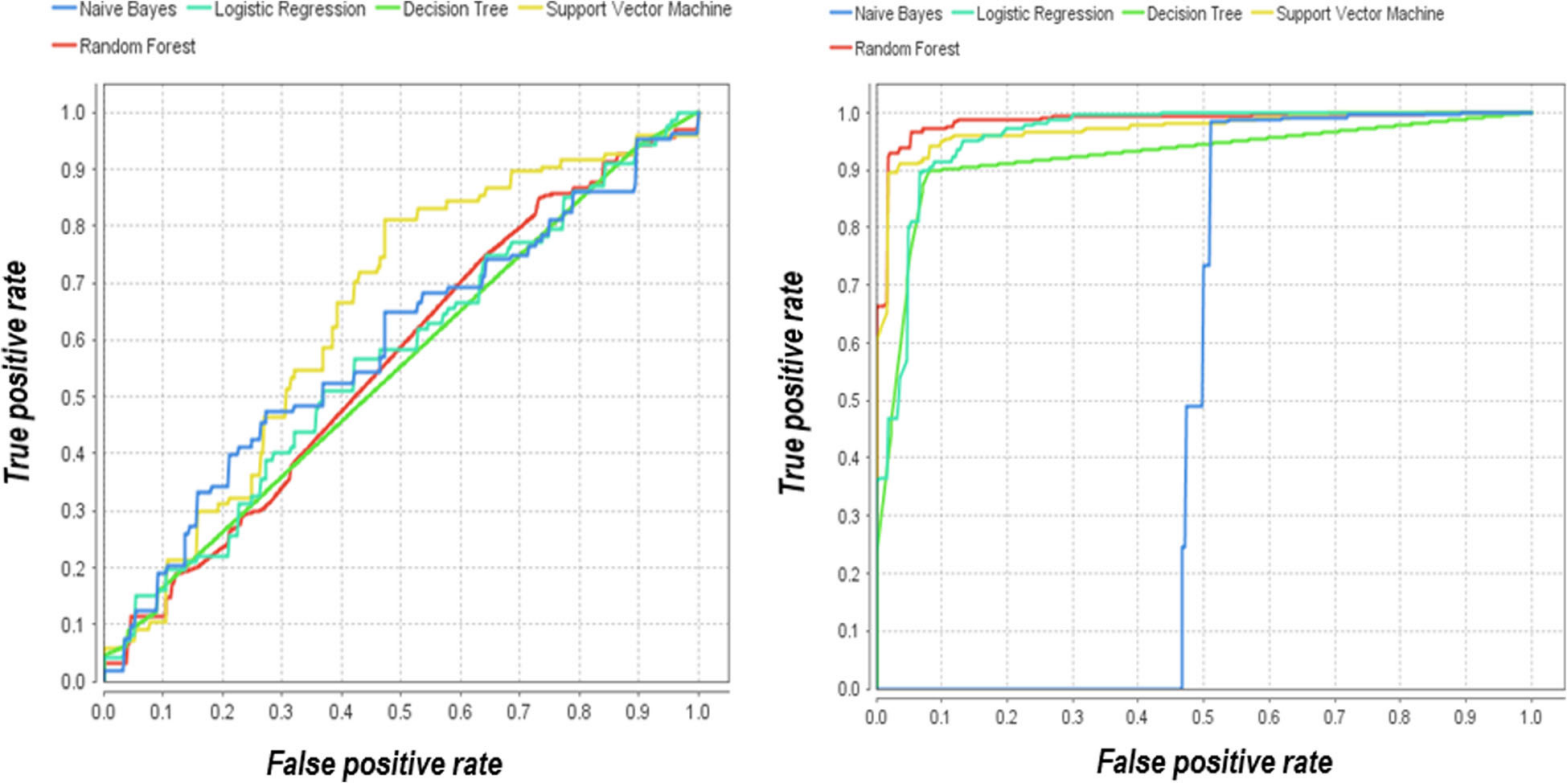
ROC compression. Graph of the error curve of classification models before and after the selection of informative descriptors based on GWO

In order to assess the quality of the binary classification, Fig. 10 presents an error curve graph (Receiver Operating Characteristic, ROC), which also allows to evaluate the effectiveness of the grey wolves optimization method for reduction of non-informative descriptors. The closer the curve is to the upper left corner, the more accurate the forecast is given by the model.

Analysis of the ROC-curve of errors shows that the prognostic ability of most algorithms increased after the selection of informative descriptors based on the grey wolves optimization method (Fig. 10). However, despite the fact that according to the Accuracy metric for the naive Bayesian algorithm, the efficiency before selecting informative descriptors is 56.9% and after 69.6%, according to the AUC indicator, this algorithm shows the least predictive result and is not effective. Therefore, on the example of researches of the author’s database of sulfanilamide descriptors under consideration, the naive Bayesian algorithm shows the worst result.

**Fig. 11:**
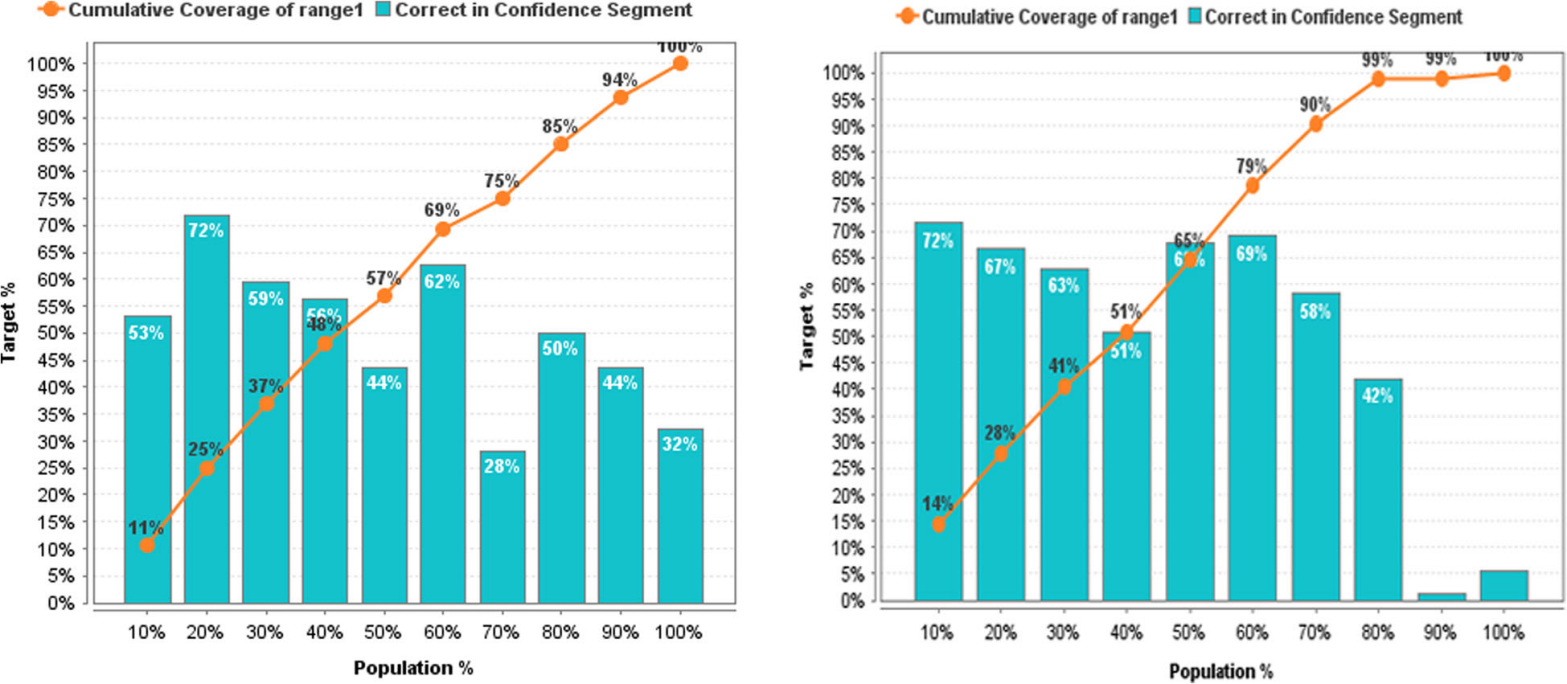
Lift Chart for Naïve Bayes. Lift diagram for evaluating the quality of forecasting of the Naïve Bayes model DB of sulfonamide descriptors before and after the preliminary data processin

**Fig. 12:**
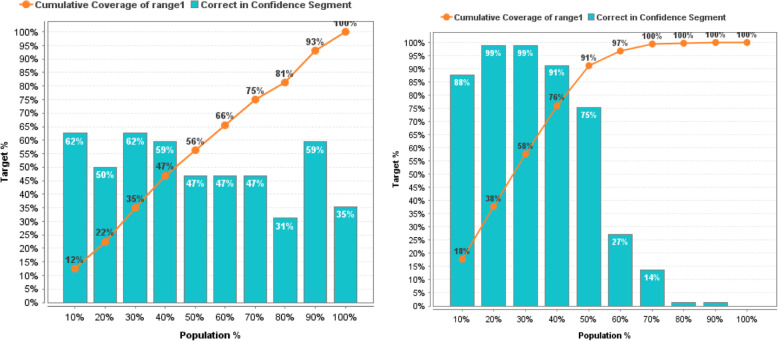
Lift Chart for Logistic Regression. Lift diagram for evaluating the quality of forecasting the Logistic Regression model of the sulfonamide descriptor database before and after the preliminary data processing

**Fig. 13:**
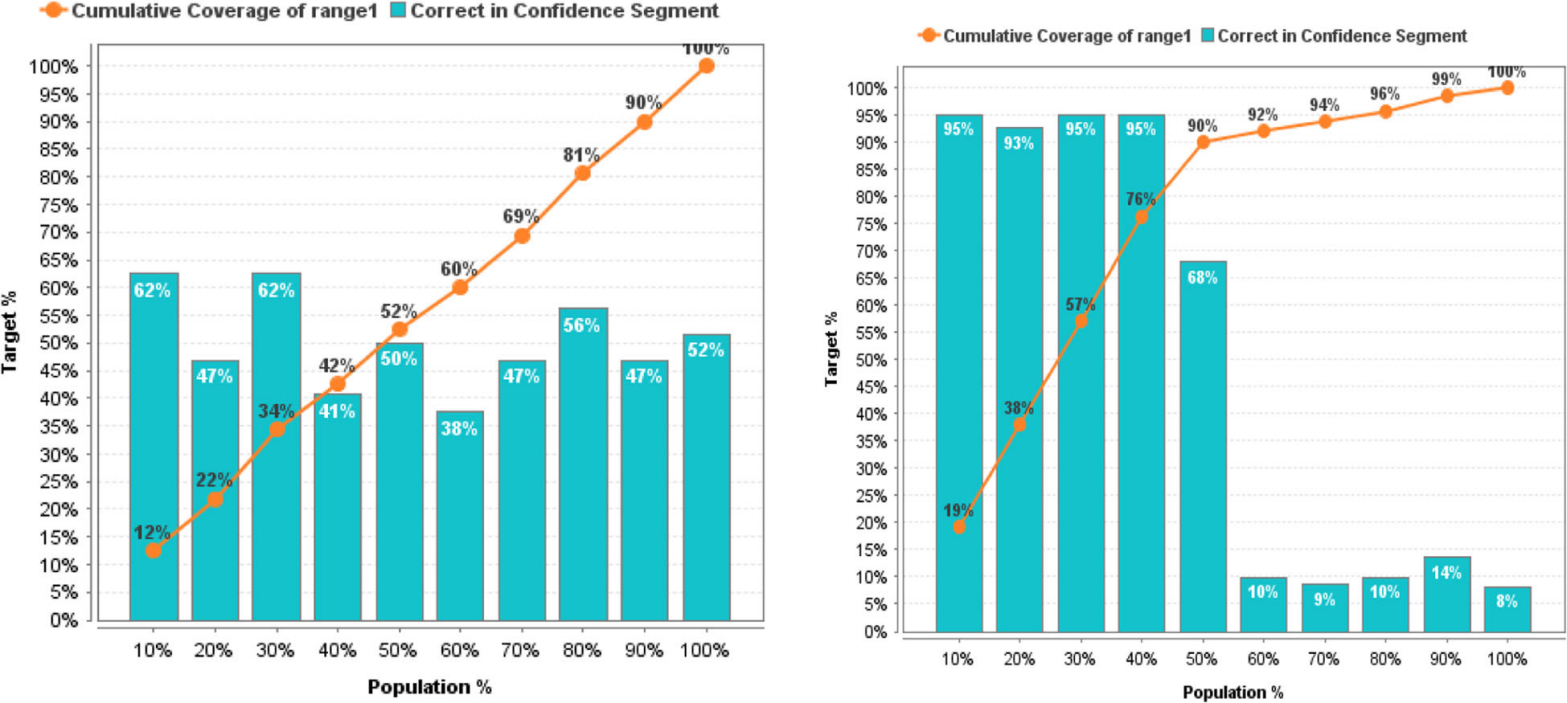
Lift Chart for Decision Tree. Lift diagram for evaluating the quality of forecasting the Decision Tree model of the sulfonamide descriptor database before and after the preliminary data processing

**Fig. 14:**
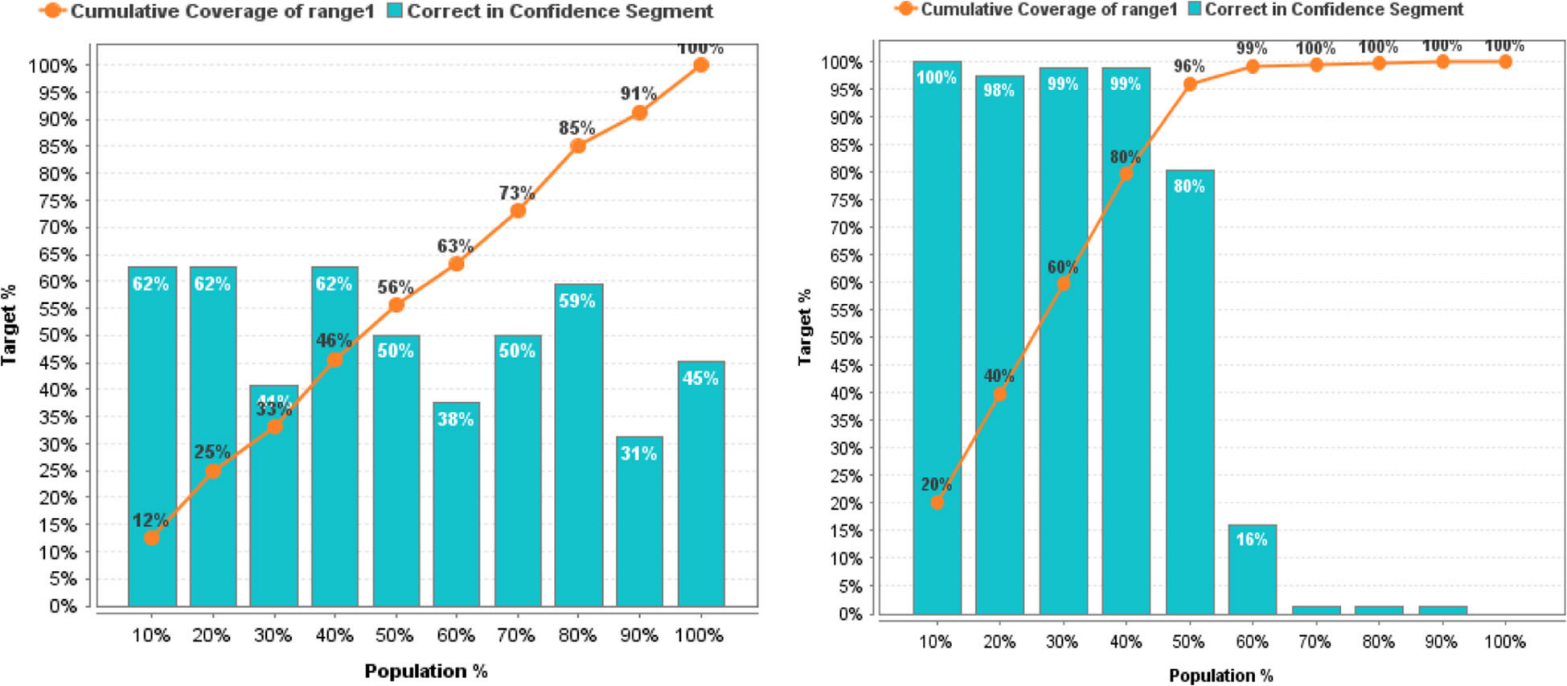
Lift Chart for Random Forest. Lift diagram for assessing the quality of forecasting the Random Forest model of the sulfonamide descriptor database before and after the preliminary data processing

**Fig. 15:**
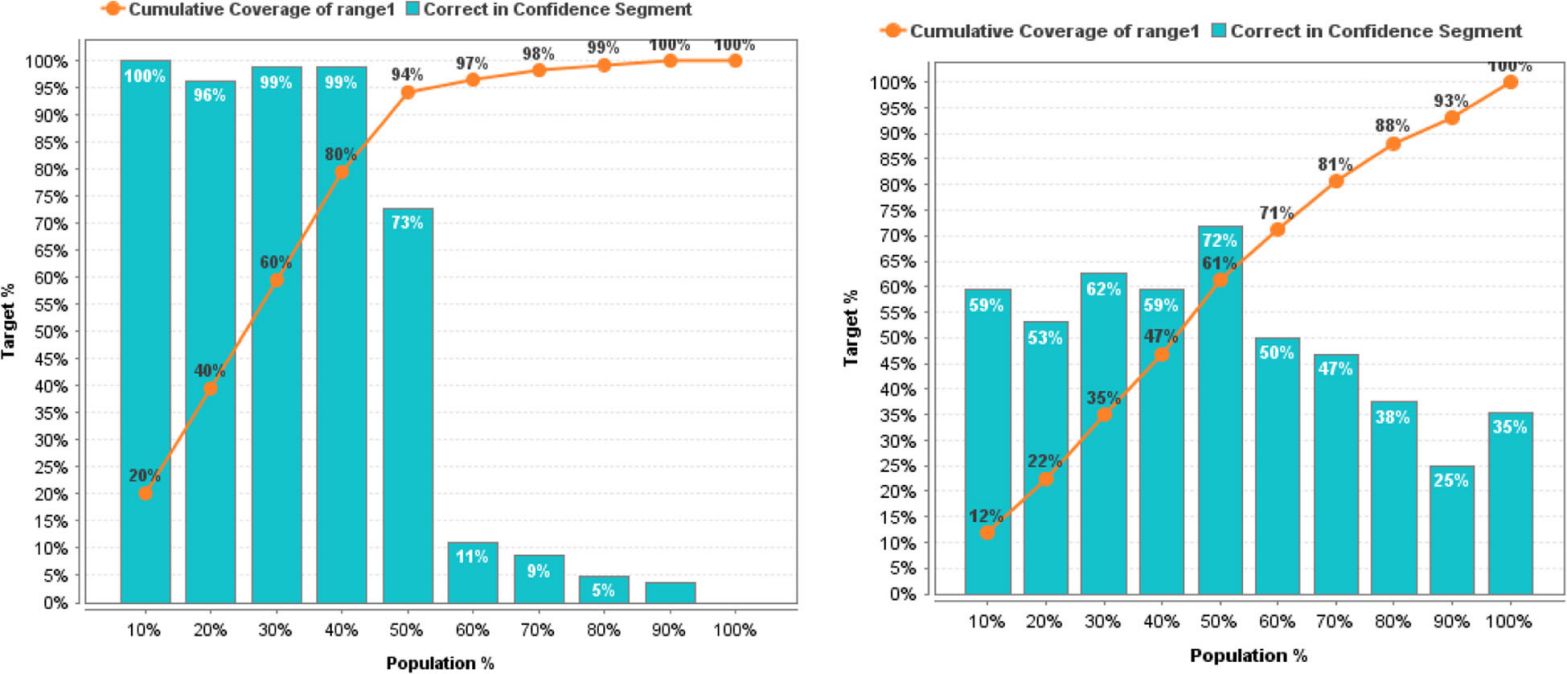
Lift Chart for Support Vector Machine. Lift diagram for evaluating the quality of forecasting the Support Vector Machine model of the sulfonamide descriptor database before and after the preliminary data processing

In more detail, the effectiveness of prediction models before and after the reduction of non-informative descriptors based on GWO can be estimated based on the lift chart presented on Figs. 11, 12, 13, 14, 15 for each algorithm, respectively.

The lift diagram allows to evaluate how much better the prediction model works compared to the random assumption (Rapid Miner). The diagram consists of two parts, the columns show the correct percentage of the target class, and the second part of the graph shows the total coverage of the target class. A feature of the diagram is that you can see the point at which predictions become less effective. Comparing the readings of the lift diagram for the prediction models under consideration, it is possible to determine which model is better, which is an additional characteristic for a deeper evaluation of the prognostic ability of algorithms in order to effectively make decisions for the selection of candidate drug compounds.

**Fig. 16:**
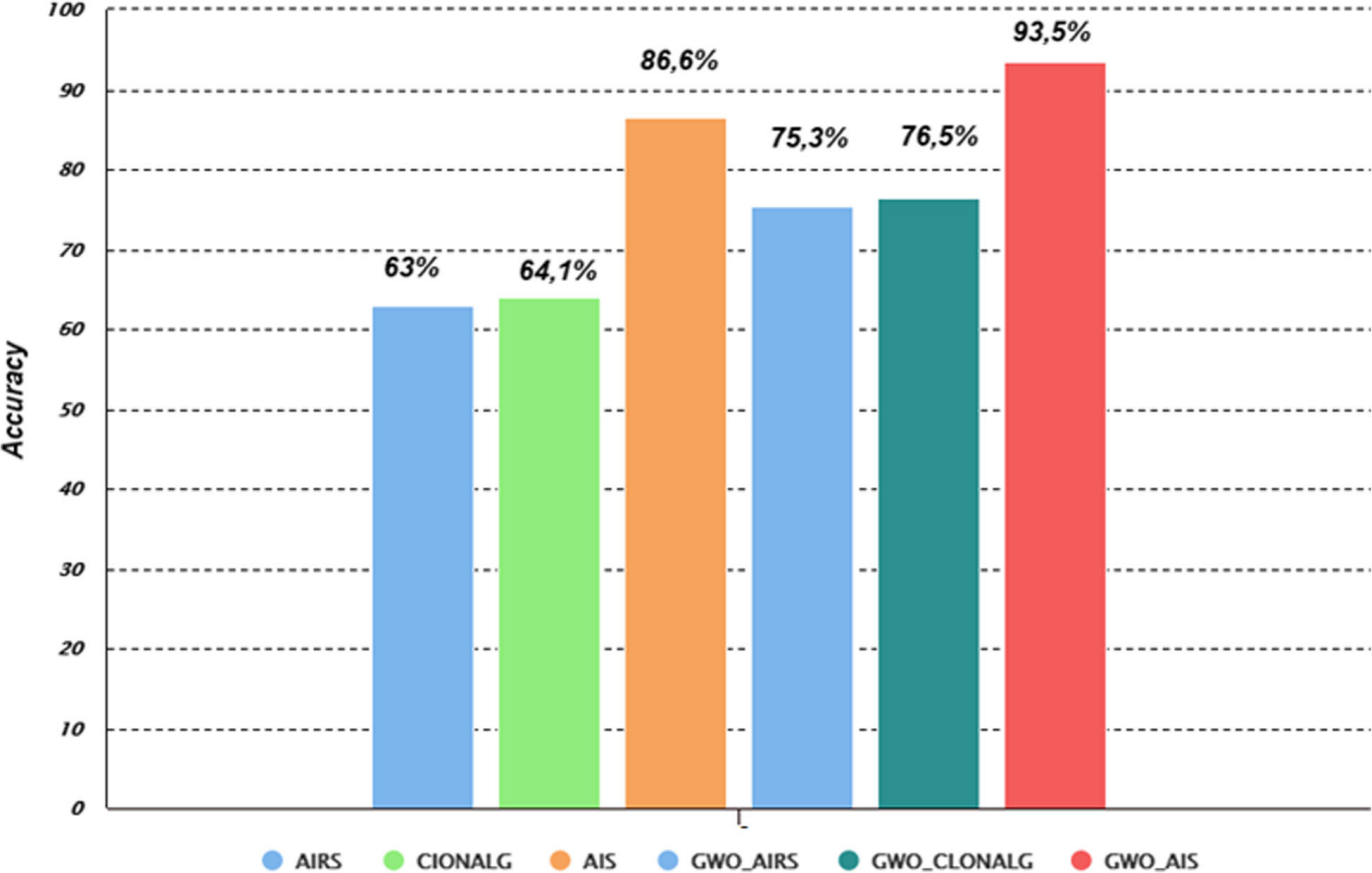
Accuracy for AIS. Efficiency of solving the classification problem based on algorithms of artificial immune systems

Therefore, the optimal database of sulfonamide descriptors can be used to predict the “structure-property” dependence of medicinal compounds. The task of classifying sulfonamides with different durations of action (Fig. 16) was carried out on the basis of several algorithms of artificial immune systems: Artificial Immune Recognition Systems (AIRS); clonal selection (CLONALG), immune network modeling (Artificial Immune System, AIS).

Below there is a comparative analysis of the effectiveness of various algorithms, which showed that the AIS models after the reduction of non-informative descriptors based on GWO give the best prognostic results: GWO-AIRS 75.3%, GWO-CLONALG 76.5% and the most effective modified algorithm is GWO - AIS 93.5% [43].

## Discussion and conclusions

The widespread and unjustified use of antibiotics leads to the ineffectiveness of the use of medicinal compounds and to the development of various side effects in the form of allergies, etc. Therefore, the urgent task is the creation of new highly effective medicinal compounds with desired properties.

Thus, the development of the theoretical foundations of computer molecular design of new antiseptic drugs - sulfonamides based on modern intellectual methods, multi-agent technology and ontological approach allows to investigate QSAR dependencies, better to understand the relationships and mechanisms of functioning of the developed multi-agent Smart-system of scientific research, helps to create efficient modified algorithms for chemical data processing and facilitates software implementation. The development of this Smart-system using ontological models allows taking into account the peculiarities of functioning and interconnections, reduces the time and computational resources at developing new drugs.

Since prediction results largely depend on the nature of the initial data (data sample size, outliers, class imbalances, etc.), currently there are no universal algorithms capable of showing high efficiency on various data sets. The developed multi-agent Smart-system based on the multi-algorithmic approach allows the use of statistical and bio-inspired methods, as well as modified algorithms based on them, in order to select models with the best predictive result. According to the results of the comparative analysis presented in the current work, the modified algorithm of artificial immune systems GWO-AIS based on the method of grey wolves optimization and immune network modeling shows high efficiency.

The advantages of using modified algorithms of artificial immune systems based on molecular recognition in a multi-agent Smart-system are: the ability of AIS to recognize patterns at the boundary of nonlinear classes (for example, when drug compounds differ structurally very slightly, but have completely different properties); as a mathematical model there can be considered a time series composed of informative descriptors for the creation of an optimal immune network model; the presence of memory; ability to self-organize and parallel data processing.

## Data Availability

The datasets generated and/or analyzed in this research can be reproduced using the computer and mathematical procedures explained in section Methods.

## Abbreviations

AIS: Artificial immune systems
AIRS: Artificial immune recognition systems
ACO: Ant colony optimization
BA: Bee algorithm
CSA: Clonal selection algorithm
DNN: Deep neural networks
GA: Genetic algorithms
GWO: Gray wolf optimization algorithm
INM: Immune network modeling
MAS: Multi-agent system
MSHCSA: Modified combinatorial recombination and adaptive mutation clonal selection algorithm
MLR: Multiple linear regressions
NN: Neural networks
NS: Negative selection
OMR: Ontological model of image recognition
PSO: Particle swarm algorithm
PCA: Principal component analysis method
QSAR: Quantitative structure-activity relationship
QSPR: Quantitative structure-property relationship
CSA: Quantum clonal selection algorithm
RF: Random forest
RSK: Ribosomal S6 kinase
ROC: Receiver Operating Characteristic
SI: Swarm intelligence
